# Urinary Cytokines Reflect Renal Inflammation in Acute Tubulointerstitial Nephritis: A Multiplex Bead-Based Assay Assessment

**DOI:** 10.3390/jcm10132986

**Published:** 2021-07-04

**Authors:** Laura Martinez Valenzuela, Juliana Draibe, Oriol Bestard, Xavier Fulladosa, Francisco Gómez-Preciado, Paula Antón, Ernest Nadal, Maria Jové, Josep Maria Cruzado, Juan Torras

**Affiliations:** 1 Nephrology Unit, Bellvitge University Hospital, Bellvitge Biomedical Research Institute (IDIBELL), Hospitalet de Llobregat, 08907 Barcelona, Spain; lmartinezv@bellvitgehospital.cat (L.M.V.); obestard@bellvitgehospital.cat (O.B.); xfulladosa@bellvitgehospital.cat (X.F.); fgomezp@bellvitgehospital.cat (F.G.-P.); panton@bellvitgehospital.cat (P.A.); jmcruzado@bellvitgehospital.cat (J.M.C.); 15268jta@comb.cat (J.T.); 2 Clinical Sciences Department, Barcelona University, Hospitalet de Llobregat, 08907 Barcelona, Spain; 3 Department of Medical Oncology, Catalan Institute of Oncology, Clinical Research in Solid Tumors (CReST) Group, Oncobell Program, IDIBELL, L’Hospitalet, 08907 Barcelona, Spain; esnadal@iconcologia.net (E.N.); mjove@iconcologia.net (M.J.)

**Keywords:** acute tubulointerstitial nephritis, T lymphocytes, biomarkers, urinary cytokines, inflammation

## Abstract

Background: Acute tubulointerstitial nephritis (ATIN) diagnosis lays on histological assessment through a kidney biopsy, given the absence of accurate non-invasive biomarkers. The aim of this study was to evaluate the accuracy of different urinary inflammation-related cytokines for the diagnostic of ATIN and its distinction from acute tubular necrosis (ATN). Methods: We included 33 patients (ATIN (*n* = 21), ATN (*n* = 12)), and 6 healthy controls (HC). We determined the urinary levels of 10 inflammation-related cytokines using a multiplex bead-based Luminex assay at the time of biopsy and after therapy, and registered main clinical, analytical and histological data. Results: At the time of biopsy, urinary levels of I-TAC/CXCL11, CXCL10, IL-6, TNFα and MCP-1 were significantly higher in ATIN compared to HC. A positive correlation between the extent of the tubulointerstitial cellular infiltrates in kidney biopsies and the urinary concentration of I-TAC/CXCL11, MIG/CXCL9, CXCL10, IL17, IFNα, MCP1 and EGF was observed. Notably, I-TAC/CXCL11, IL-6 and MCP-1 were significantly higher in ATIN than in ATN, with I-TAC/CXCL11 as the best discriminative classifier AUC (0.77, 95% CI 0.57–0.95, *p* = 0.02). A combinatory model of these three urinary cytokines increased the accuracy in the distinction of ATIN/ATN compared to the individual biomarkers. The best model resulted when combining the three cytokines with blood eosinophil and urinary leukocyte counts (LR = 9.76). Follow-up samples from 11ATIN patients showed a significant decrease in I-TAC/CXCL11, MIG/CXCL9 and CXCL10 levels. Conclusions: Urinary I-TAC/CXCL11, CXCL10, IL6 and MCP-1 levels accurately distinguish patients developing ATIN from ATN and healthy individuals and may serve as novel non-invasive biomarkers in this disease.

## 1. Introduction

Acute tubulointerstitial nephritis (ATIN) is a prevalent cause of immune-mediated acute kidney injury (AKI), accounting for 15–20% of cases [1]. Frequently ATIN courses as a type IV hypersensitivity reaction against a long list of drugs [2], such as non-steroidal anti-inflammatory drugs (NSAIDs), various antibiotics and proton-pump inhibitors (PPIs). The immune checkpoint inhibitor (ICI) is an emerging cause of ATIN although its exact pathogenic mechanism is still uncertain. In ICI-associated ATIN, the loss of peripheral tolerance to previously tolerated renal antigens or drugs, or an ICI off-target effect over the tubular epithelial cells (TECs) may be the underlying causes. In addition, some systemic diseases and infections can be associated with ATIN too. In all cases of ATIN immune cells exert direct cytotoxicity over TECs and release proinflammatory cytokines. Therefore, TECs acquire an active role in ATIN due to their antigen presenting cell ability and their production of chemokines and signaling molecules related to immune cell recruitment [3].

The gold standard for ATIN diagnostic is kidney biopsy. The main pathologic lesion is a T-cell tubulointerstitial infiltrate occasionally accompanied by eosinophils, macrophages or plasma cells [4]. Reliable non-invasive biomarkers for the diagnosis and follow-up are lacking. As in other kidney diseases, clinicians have focused on urine to find ATIN biomarkers. For years it was assumed that eosinophiluria was a good ATIN biomarker, but Muriithi et al. described that eosinophiluria had only 31% sensitivity and 68% specificity for ATIN diagnosis. Moreover, sterile leukocyturia prevalence ranged between 50% and 70% and white blood cell casts are unspecific, also showing up in glomerulonephritis and pyelonephritis [5]. As in ATIN, a tubular dysfunction portrait with mild proteinuria and preserved urine output is often observed in acute tubular necrosis (ATN) and the distinction between these diseases results is challenging. Unfortunately, there are no reliable biomarkers to discriminate between ATIN and ATN [6].

The aim of this study was to evaluate the accuracy of a panel of 10 urinary cytokines, for ATIN diagnosis and in the distinction of this disease from ATN. We also sought to provide a rationale for their implementation in the clinical practice.

## 2. Material and Methods

### 2.1. Experimental Design and Study Population

This is an observational study. All patients diagnosed and prospectively followed at Bellvitge University Hospital between January 2018 and June 2020, with biopsy-proven ATIN were eligible for the study. All patients with the diagnosis of ATIN were treated with prednisone at a 1 mg/Kg weight with slow tapering over the subsequent weeks. In the same study period, a cohort of patients diagnosed of ATN by kidney biopsy were recruited as an acute tubular disease comparator. Exclusion criteria were as follows: documented urinary tract infection, sepsis, or evidence of superimposed glomerular disease. Healthy controls were volunteers from the institution without any known medical condition or pharmacological treatment. All patients signed informed consent prior to their recruitment. The Ethical Committee of Bellvitge University Hospital approved the study protocol (PR143/19) on 9 May 2019.

### 2.2. Clinical Variables

At baseline, we registered the main demographic variables of interest: age, sex, etiology of the ATIN/ATN episode. We also registered analytical parameters at baseline and when diagnosis was made: serum creatinine, urinary leukocyte and erythrocyte count, proteinuria, blood eosinophil count, and serum C-reactive protein (CRP) levels. Those parameters were also registered when follow-up samples were obtained in selected patients.

### 2.3. Histological Grading of Tubule-Interstitial Infiltrates

Histological grading of the tubulointerstitial infiltrates was performed by the pathologist of our institution specialized in the evaluation of kidney biopsies. The inflammatory infiltrate was evaluated in non-scarred areas of the cortex and graded into the following categories depending on the extent: grade 0 (affecting <25%), grade 1 (affecting 25–50%), grade 2 (affecting 50–75%) and grade 3 (affecting >75%). The presence of granulomas and eosinophils in the inflammatory infiltrates was also registered.

### 2.4. Sampling and Measurement of Urinary Cytokine Levels

Urine samples were collected shortly after diagnosis and prior to treatment in all patients. In a selected cohort of ATIN patients a follow-up sample at least one month after diagnostic was also obtained. Urine samples were centrifuged at 2000 rpm for 10 min. Supernatant was collected and stored in 1 mL aliquots at −80 °C until cytokine determination. Based on the literature review, we selected a panel of 10 inflammatory cytokines (interferon (IFN)-α2, epidermal grow factor (EGF), interleukin (IL)-1β, IL-17A, IL-6, IFNγ-induced protein 10 (IP-10)/C-X-C motif chemokine ligand (CXCL)-10, tumor necrosis factor (TNF)α, monocyte chemoattractant protein-1 (MCP1), IFN-inducible T-cell alpha chemoatractant (I-TAC)/C-X-C motif chemokine 11 (CXCL11), monokine induced by IFNγ (MIG)/CXCL9) and customized a Multiplex Immunoassay commercial kit in the Luminex platform (Invitrogen ProcartaPlex, Thermofisher Scientific, Waltham, MA, USA) that was performed according to the manufacturer’s instructions. A Luminex MAGPIX^®®^reader was used to retrieve the results.

Urine protein concentration was assessed using a Bicinchoninic Acid (BCA) protein quantitation assay (Pierce™ BCA Protein Assay Kit, Thermofisher Scientific, Waltham, MA, USA) according to manufacturer’s instructions.

## 3. Statistical Analysis

Data was analyzed using GraphPad Prism version 6.00 (GraphPad Software, La Jolla, CA, USA) and IBM SPSS Statistics Version 20.0 (IBM corp., Armonk, NY, USA). To determine the Gaussian distribution of the variables, Kolmogorov–Smirnov test was applied. ANOVA test was used to compared the means of quantitative values among three groups, while for comparison between two groups, Student’s *t*-test or Mann–Whitney U test was used depending on the distribution of the variable. Comparison of the frequency of qualitative variables was performed using a Chi-squared test. Correlations were assessed using Spearman’s correlation. To evaluate the performance of the cytokines in the distinction between ATN and ATIN, we plotted ROC curves and calculated the AUC. The optimal cutoff was calculated according to then Youden method. The recursive partitioning method was used to perform a combinatory model of biomarkers, and the result was plotted as a Classification and Regression Tree after a 10-fold cross-validation. A Fagan nomogram was used to calculate post-positive test probabilities. *p*-Values < 0.05 were considered significant.

## 4. Results

### 4.1. Baseline Characteristics of the Population

In total, 33 patients were recruited (21 patients diagnosed with ATIN and 12 patients with ATN) and six healthy controls (HC). Baseline characteristics of the ATIN and ATN cohorts are shown in Table 1. Most of the ATIN cases were related to pharmacological treatments: ICI (50% of patients), NSAIDs (23.8%) and PPIs (19.04%). ATN cases were mainly caused by drugs such as platin (25%), NSAIDs (16%) and aminoglycosides (16%).

As shown in Table 1, ATIN and ATN patients did not differ in kidney function, urinary leukocyte count and serum CRP. Eosinophil blood count was significantly higher in ATIN patients.

### 4.2. Urinary Cytokine Levels Are Significantly Increased in ATIN Patients and Correlate with the Extend of Tubulointerstitial Infiltrate

We first performed an ANOVA analysis to determine whether there were any statistically significant differences between the means of the concentration of the different cytokines and chemokines between the three analyzed groups. We found statistically significant differences in the concentration of I-TAC/CXCL11, IL6, TNFα, MCP1 and EGF (see Table 2).

We then compared the urinary concentration noticed in patients with ATIN and healthy controls. As shown in Table 3, the urinary levels of I-TAC/CXCL11, CXCL10, IL-6, TNFα and MCP-1 were significantly higher among ATIN patients as compared to HC.

Next, the tubulointerstitial mononuclear cell infiltration observed in the kidney biopsies was graded in four categories as described in the Section 2. According to this classification, 23.5% of the patients with ATIN had Grade 1 tubulointerstitial infiltration, while the infiltrate was Grade 2 in 29.4% of cases, and 47.1% patients had Grade 3 infiltrates. Two patients showed the presence of granulomas in the kidney biopsy, and the infiltrates contained eosinophils in 12 cases.

A strong positive correlation was observed between the grade of tubulointerstitial infiltration and most urinary cytokines: I-TAC/CXCL11 (rho = 0.574, *p* = 0.001), MIG/CXCL9 (rho = 0.376, *p* = 0.049), CXCL10 (rho = 0.614, *p* = 0.001), IL17 (rho = 0.398, *p* = 0.041), IFNα (rho = 0.403, *p* = 0.034), MCP1 (rho = 0.472, *p* = 0.013), EGF (rho = 0.41, *p* = 0.03) (Supplementary Table S1). Conversely, no correlation was observed between the grade of tubulointerstitial infiltrates and main biochemical parameters, such as serum creatinine, urinary leukocyte or erythrocyte counts or blood eosinophil numbers, besides serum CRP (rho = 0.435, *p* = 0.021).

### 4.3. Urinary Cytokine Levels Distinguish ATIN from ATN Patients

**Figure 1:**
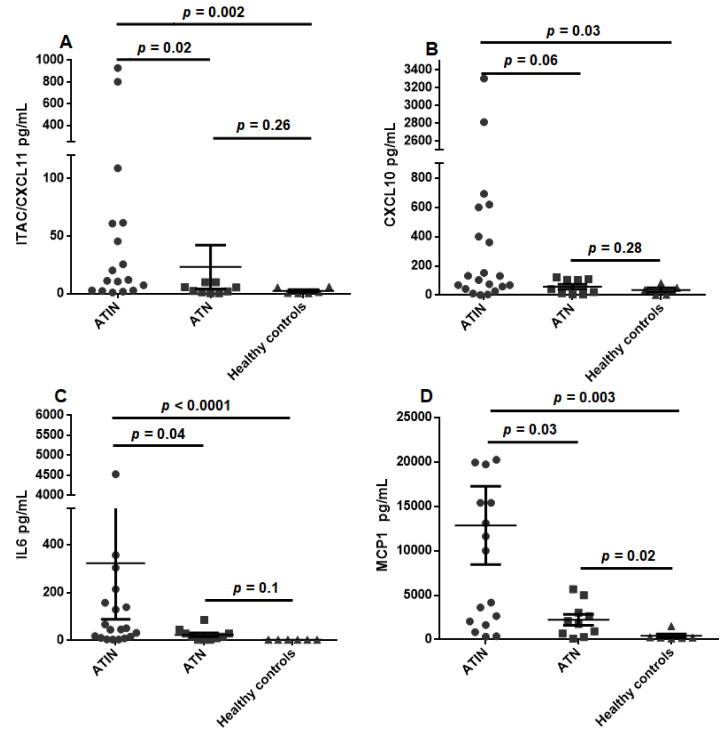
Urinary levels of CXCL11 (**A**), CXCL10 (**B**), IL-6 (**C**) and MCP-1 (**D**) in acute tubulointerstitial nephritis and acute tubular necrosis patients, and healthy controls. ATIN—acute tubulointerstitial nephirits, ATN—acute tubular necrosis.

We next compared the urinary cytokine levels between ATIN and ATN patients. As seen in Figure 1, and described in Table 4, urinary cytokine levels of I-TAC/CXCL11, IL-6 and MCP-1 were significantly higher in ATIN as compared to ATN patients. CXCL10 was also numerically higher in ATIN patients, although it did not reach statistical significance (*p* = 0.06). Notably, urinary cytokine levels between ATN patients and HC were not significantly different, but for uMCP1 (*p* = 0.022) as shown in Table 5.

To determine whether the presence of biomarkers in urine was associated with abnormal glomerular filtration barrier, we compared the ratio of urine biomarkers to urine albumin between AIN and controls. We found that this ratio was higher in AIN than in controls. Taken together, these approaches suggest that the urine biomarkers originated primarily in the kidneys.

**Figure 2:**
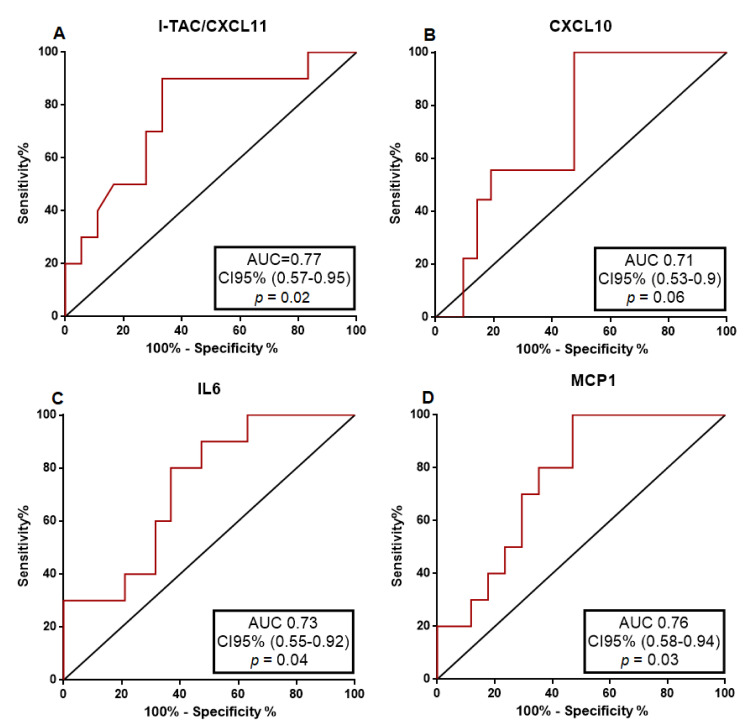
The ROC curves of CXCL11 (**A**), CXCL10 (**B**), IL-6 (**C**) and MCP-1 (**D**) in the distinction of acute tubulointerstitial nephritis from acute tubular necrosis. AUC—Area under the curve; CI95%—Confidence interval 95%.

To further investigate the predictive accuracy of each urinary cytokine distinguishing the presence of ATIN from ATN, we performed a receiver operating characteristic (ROC) curve analysis. As illustrated in Figure 2 and Table 6, most urinary cytokines displayed a good predictive capacity for ATIN with AUC higher than 0.7. Nonetheless, I-TAC/CXCL11 showed the best accuracy distinguishing between ATIN and ATN (AUC = 0.77, 95% CI 0.57–0.95), *p* = 0.02).

**Figure 3:**
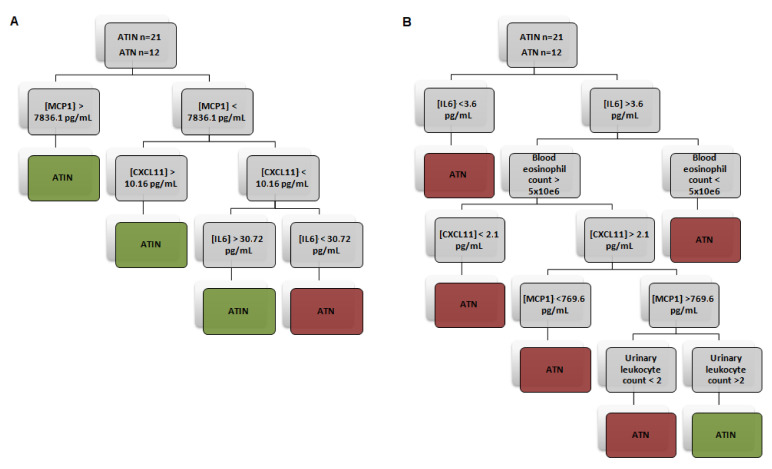
Decision trees elaborated by recursive partitioning combining different classical and novel biomarkers of acute tubulointerstitial nephritis. Model 1 (**A**) includes urinary CXCL11, MCP-1 and IL6 levels. Model 2 (**B**) combines urinary level of these cytokines with urinary leukocyte and blood eosinophil counts. ATIN—acute tubulointerstitial nephirits, ATN—acute tubular necrosis.

To maximize the performance of individual biomarkers in the classification of the patients into ATIN and ATN categories, we constructed several models using the recursive partitioning method to plot a decision tree. In Model 1, we found that gathering urinary uCXCL11, MCP1 and IL-6 were the best matched to define patients into ATIN or ATN categories. Separately, these biomarkers showed a rather high specificity and PPV, but lower sensitivity and NPV. Conversely, their combination increased sensitivity to 84.2% and NPV to 76.9%, while maintaining a high specificity (83.3%) and PPV (88.9%), and increasing the likelihood ratio (LR) up to 5.04. Post-positive test probability, that is, the probability of having the target condition if the test falls out positive using a Fagan nomogram, was 88.4% when using Model 1. Thereafter, we sought to combine the three novel biomarkers with the classical ATIN biomarkers. The highest accuracy was obtained combining the three biomarkers with the blood eosinophil count and the urinary leukocyte count (Model 2). This Model 2 almost doubled the positive LR of Model 1 and increased it up to 9.76. Applying Model 2, we found a post-positive test probability of 94.5% in our cohort (see Figure 3 and Table 6).

### 4.4. Urinary Cytokine Levels Decrease after Treatment and Recovery of Renal Function

**Figure 4:**
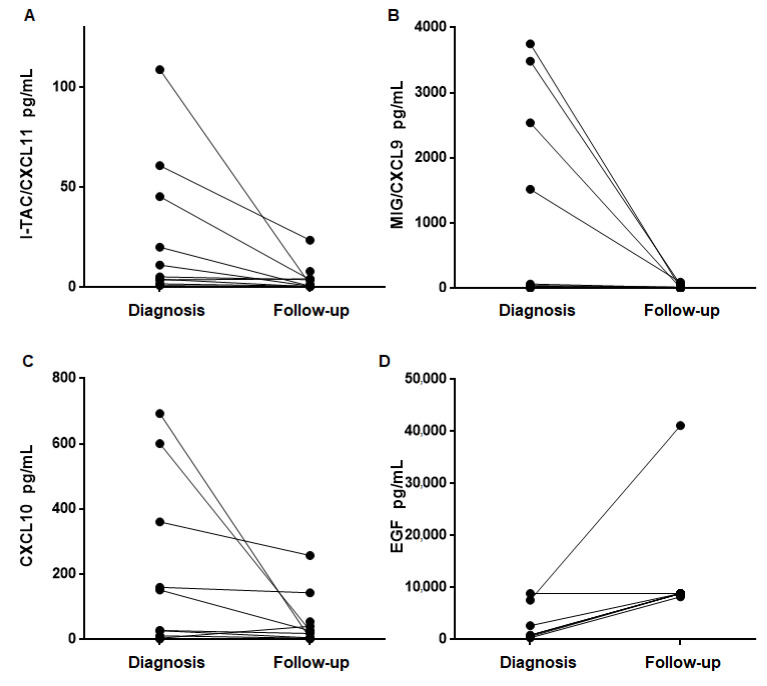
The urinary concentration of CXCL 11 (**A**), CXCL9 (**B**), CXCL10 (**C**) and EGF (**D**) at the moment of the diagnosis of acute tubulointerstitial nephritis and in the follow-up after treatment.

In all patients, kidney function improved at the time of the follow-up assessment at least one month after prednisone initiation in all the patients (serum creatinine at time of diagnosis 291.7 ± 47.67 µmol/L vs. serum creatinine at time of follow-up 123.8 ± 13.09 µmol/L *p* = 0.002). We next compared the concentration of all cytokines between the time of diagnosis and at the follow-up visit. Follow-up samples were available in 11/21 (52.3%) of ATIN patients. As illustrated in Figure 4, I-TAC/CXCL11, MIG/CXCL9 and CXCL10 levels significantly dropped in follow-up samples as compared to diagnosis samples reaching the same levels as HC. IL6, IL17 and TNFα levels also decreased but without reaching statistical differences. Interestingly, EGF levels increased in follow-up. As compared to diagnostic samples (*p* = 0.016). Supplementary Table S2 shows the value of cytokines at the time of both diagnosis and follow-up. Figure 4 depicts the cytokine plots before and after treatment, with a significant reduction in follow-up samples, and an increase in the case of EGF.

## 5. Discussion

ATIN is a prevalent cause of AKI among hospitalized patients and, usually, it is difficult to distinguish from conventional ATN, which is also a frequent cause of AKI. Both diseases share clinical features and analytical findings given the presence of tubular dysfunction and can be related to multiple pharmacological treatments as a common risk factor. ATIN is an immune-mediated condition, as the infiltration of immune cells at the tubulointerstitial space is the histological hallmark of the disease, which is only mild or even absent in the case of ATN. Based on the pathophysiology of the disease, and on data from previous works of our group in the setting of kidney transplantation where we assessed CXCL9 [7], we examined the urinary concentration of a panel of 10 cytokines related to inflammation in order to discover new biomarkers of ATIN which could in turn help in the clinical distinction between ATIN and ATN.

Sterile leukocyturia has been used classically to evidence the presence of kidney inflammation, but has low sensitivity as it is only generally found in 50–70% of patients depending on the series [8]. Inflammatory markers as CRP have also been reported to be elevated in ATIN in association with the presence and intensity of the inflammatory burden of the cellular infiltrates [9]. In our study, sterile leukocyturia and serum CRP levels were not different between ATIN and ATN patients. The only classical parameter that showed significant differences between the two cohorts was blood eosinophil count, in line with the immune-allergic basis of ATIN. Our findings confirm that the study of the urinary sediment is suboptimal in the diagnosis of ATIN and support the necessity of new biomarkers related to the pathophysiology of the disease.

In our study, we first found that the urinary concentration of I-TAC/CXCL11, CXCL10, IL6, TNF-α and MCP-1 was significantly higher in ATIN patients as compared to healthy controls. The CXCL9-CXCL10-CXCL11 axis is involved in immune cell migration promoting the establishment of chemotactic gradients, immune cell differentiation, Th1 polarization and activation of lymphocytes [10]. TNF-α is a strong stimulus for the production of the cytokines of this axis [11]. IL-6 stimulates T-cell proliferation and resistance to apoptosis, as well as the release of Th2 cytokines. TECs produce IL-6 in response to local insults. In turn, IL-6 promotes collagen I production by TECs thus accelerating tubulointerstitial fibrosis [12]. MCP-1 is also a key protein in the recruitment of monocytes, neutrophils, and lymphocytes in damaged tissues. As in the case of IL-6, TECs can produce MCP-1 but also the endothelial cells of the peritubular capillaries and macrophages can do so themselves. Higher urinary levels of MCP-1 and IL-6 in ATIN patients found in our study are consistent with the findings reported by other authors [13,14]. In addition to higher urinary levels of MCP-1, Chen et al. [13] also found higher levels of serum TNFα and IL6 in ATIN patients compared to healthy controls, thus suggesting a role of the cytokines in this disease, but they did not evaluate their urinary concentration. In contrast, Moledina et al. found significantly higher urinary levels of TNFα and IL-9 in ATIN compared to ATN patients, and suggested two cutoffs (a highly sensitive and a highly specific cutoff) to define which patients should not require a kidney biopsy for diagnosis [15,16]. In addition to higher IL-6 levels, Yun et al. demonstrated higher urinary levels of IFNα and IL17A in ATIN patients compared to healthy controls, but we were not able to confirm these findings in our cohort [14]. In contrast, healthy controls exhibited higher EGF urinary levels compared to ATIN patients. EGF, produced also by TECs, participates in the maintenance of the integrity of the renal tubular epithelium [17] and lower urinary levels have been described in the occurrence of AKI and suggested as a biomarker by some authors [18]. Our study provides new evidence that the elevation of other three cytokines with a clear biological significance based on their known role in inflammation (CXCL11, CXCL10 and TNF-α) are actively present in the urine of ATIN patients.

In addition, we found a positive correlation between the extent of the tubulointerstitial cellular infiltrate in kidney biopsies with the urinary concentration of I-TAC/CXCL11, MIG/CXCL9, CXCL10, IL17, IFNα, MCP1 and EGF. This association indicates the local production of these cytokines in the kidney during the acute inflammatory process and their release to the urine. This finding is of great importance as it does not only reflect the pathophysiology of the disease itself, but it also reflects the extent of the inflammatory damage in the kidneys, information that with current available clinical tools can only be assessed through a direct histological assessment after a kidney biopsy.

A second important finding of our work is that urinary levels of I-TAC/CXCL11, IL6 and MCP-1 were significantly higher in ATIN as compared to ATN patients, with barely significant CXCL10 levels. Notably, in a ROC curve analysis, CXCL-11 showed the best discriminative performance in the distinction of ATIN from ATN, based on its greater AUC and LR. We were able to increase the discrimination performance of the biomarkers by the elaboration of a combinatory model using both the three urinary cytokines and some of the clinical parameters such as blood eosinophils and urinary leukocyte counts, which revealed a high post-positive test probability of up to 94.5%. Other authors used combination of biomarkers in order to enhance diagnostic reliability in other diseases [19,20], but to the best of our knowledge this is the first study that applies this statistical strategy in ATIN and ATN discrimination. The proposed urinary biomarkers were more specific than sensitive, but the combinatory model of the three cytokines allowed a significant increase in the sensitivity of the test. We think that this elevated post-positive test probability is adequate to diagnose and could help in taking therapeutic decisions in the case of unavailability or contraindication of kidney biopsy.

Finally, we also assessed the kinetics of these urinary cytokines over time after treatment in a subset of patients with ATIN. Interestingly, we found a normalization of the concentration of the cytokines in ATIN patients after treatment reaching similar levels to those observed among healthy individuals. In particular, CXCL9/CXCL10/CXCL11 showed the most significant decrease in the follow-up samples. This axis controls the attraction of immune cells to damaged sites, and its cessation may reflect the resolution of the inflammatory process. In the field of kidney transplant, our group and others [21] showed significantly increased levels of uCXCL9 and uCXCL10 in patients undergoing acute T-cell mediated rejection as compared to pristine allografts, and furthermore, these urinary cytokines could also help discriminating those patients with good response to rescue therapies [7,22]. Notably, to our knowledge, this is the first report showing the presence of high CXCL9/CXCL10/CXCL11 urinary levels in ATIN. Interestingly, unlike the other evaluated cytokines, urinary EGF levels significantly increased in follow-up samples, a finding that strongly suggests that these patients were undergoing a progressive recovery of the tubular epithelium after the inflammatory injury.

Our study has some limitations. First, while we described a high discriminatory accuracy for patients with ATIN by developing distinct statistical models, these data should be validated in a larger independent patient cohort for confirmatory results. However, the high post-positive test probability observed (higher than 90%), strongly suggests the consistency of our data and counterbalance this constraint. Moreover, the low number of patients that were followed-up and evaluated may be also considered as a limitation. Nonetheless, the consistently low urinary levels observed in all evaluated patients underscores the value of these data. Finally, the low specificity of these cytokines for ATIN may be considered as another limitation but applied in the adequate clinical setting—presence of AKI with a tubular dysfunction profile, excluding infections and other glomerular disease features—it has a high accuracy to differentiate between inflammation and tubular dysfunction in the setting of ATN.

In conclusion, the higher levels of these specific cytokines in ATIN compared to ATN patients and healthy controls, and the significant decline after treatment allow us to propose I-TAC/CXCL11, CXCL10, IL6 and MCP-1, alone or in combination, as candidate cytokines that may serve as novel diagnostic and follow-up biomarkers in patients undergoing AKI due to ATIN. The usefulness of these biomarkers is supported by their biological significance and their role in inflammatory processes. The non-invasive nature of these tests is of the utmost importance, as it may allow frequent monitoring, as a dynamic assessment of the evolution of the disease. The generalization of bead-based assays that allow the determination of large panels of different cytokines in a more efficient way opens a wide field with clear benefits applicable to clinical practice.

## Figures and Tables

**Table 1:** Baseline characteristics of the ATIN and ATN cohorts. * Statistically significant differences. CRP—C reactive protein. ATIN—acute tubulointerstitial nephritis; ATN—acute tubular necrosis.

	ATIN (*n* = 21)	ATN (*n* = 12)	*p*-Value
Age (years)	66.5 ± 13.52	63.5 ± 12.5	0.536
Sex (%male)	57.10%	81.80%	0.248
Diabetes (% patients)	33.3%	41.66%	0.365
Hypertension (% patients)	57.14%	50%	0.432
Baseline creatinine (µmol/L)	87.84 ± 19.17	89.58 ± 15.56	0.322
Creatinine (µmol/L)	324.9 ± 141.8	321.7 ± 176.3	0.956
CRP (mg/L)	74.9 ± 95.1	25.6 ± 59.7	0.129
Urinary leukocyte count (leukocytes/ μL)	400 ± 1467	166.5 ± 452.7	0.613
Urinary erythrocyte count (leukocytes/ μL)	248.6 ± 1075.5	46.8 ± 80.1	0.542
Proteinuria (g/mol)	70.49 ± 114.9	94.8 ± 112.5	0.587
Eosinophil blood count (×10^6^)	257.2 ± 210.7	102.7 ± 105.4	0.03 *

**Table 2:** ANOVA test comparing the urinary concentration of the evaluated cytokines in patients with ATIN, ATN, and healthy controls. * Statistically significant differences. ATIN—acute tubulointerstitial nephritis; ATN—acute tubular necrosis.

	ATIN (*n* = 21)	ATN (*n* = 12)	Healthy Controls (*n* = 6)	*p*-Value
I-TAC/CXCL11	129.5 ± 64.68	23.07 ± 19.01	2.35 ± 1.07	0.046 *
MIG/CXCL9	1170 ± 739.5	1456 ± 896.2	43.37 ± 9.91	0.665
IL1B	9.08 ± 5.84	14.56 ± 9.73	2.03 ± 0.53	0.241
CXCL10	909.9 ± 468.1	58.60 ± 16.74	35.31 ± 15.3	0.105
IL6	323.1 ± 235.1	24.09 ± 8.11	3.2 ± 0.2	0.029 *
IL17	7.28 ± 5.15	4.67 ± 2.37	2.18 ± 0.65	0.83
TNFα	5.46 ± 1.12	4.17 ± 0.89	2.84 ± 0.27	0.032 *
IFNα	23.04 ± 13.82	18.21 ± 6.99	27.48 ± 13.8	0.669
MCP1	12848 ± 4413	2221 ± 603.2	418 ± 217.6	0.002 *
EGF	3888 ± 851.3	3067 ± 1207	18748 ± 6454	0.003 *

**Table 3:** Comparison of the urinary concentration of the evaluated cytokines in patients with ATIN and healthy controls. * Statistically significant differences. ATIN—acute tubulointerstitial nephirits.

	ATIN (*n* = 21)	Healthy Controls (*n* = 6)	*p*-Value
I-TAC/CXCL11	129.5 ± 64.68	2.35 ± 1.07	0.002 *
MIG/CXCL9	1170 ± 739.5	43.37 ± 9.91	0.629
IL1B	9.08 ± 5.84	2.03 ± 0.53	0.693
CXCL10	909.9 ± 468.1	35.31 ± 15.3	0.034 *
IL6	323.1 ± 235.1	3.2 ± 0.2	<0.001 *
IL17	7.28 ± 5.15	2.18 ± 0.65	0.781
TNFα	5.46 ± 1.12	2.84 ± 0.27	0.027 *
IFNα	23.04 ± 13.82	27.48 ± 13.8	0.558
MCP1	12848 ± 4413	418 ± 217.6	0.001 *
EGF	3888 ± 851.3	18748 ± 6454	0.002 *

**Table 4:** Comparison of the urinary concentration of the evaluated cytokines in patients with ATIN and ATN. * Statistically significant differences. ATIN—acute tubulointerstitial nephirits, ATN—acute tubular necrosis.

	ATIN (*n* = 21)	ATN (*n* = 12)	*p*-Value
I-TAC/CXCL11	129.5 ± 64.68	23.07 ± 19.01	0.022 *
MIG/CXCL9	1170 ± 739.5	1456 ± 896.2	0.945
IL1B	9.08 ± 5.84	14.56 ± 9.73	0.199
CXCL10	909.9 ± 468.1	58.60 ± 16.74	0.060
IL6	323.1 ± 235.1	24.09 ± 8.11	0.043 *
IL17	7.28 ± 5.15	4.67 ± 2.37	0.664
TNFα	5.46 ± 1.12	4.17 ± 0.89	0.300
IFNα	23.04 ± 13.82	18.21 ± 6.99	0.473
MCP1	12848 ± 4413	2221 ± 603.2	0.026 *
EGF	3888 ± 851.3	3067 ± 1207	0.386

**Table 5:** Comparison of the urinary concentration of the evaluated cytokines in patients with ATN and healthy controls. * Statistically significant differences. ATN—acute tubular necrosis.

	ATN (*n* = 12)	Healthy Controls (*n* = 6)	*p*-Value
I-TAC/CXCL11	2 3.07 ± 19.01	2.35 ± 1.07	0.269
MIG/CXCL9	1456 ± 896.2	43.37 ± 9.91	0.732
IL1B	14.56 ± 9.73	2.03 ± 0.53	0.110
CXCL10	58.60 ± 16.74	35.31 ± 15.3	0.298
IL6	24.09 ± 8.11	3.2 ± 0.2	0.105
IL17	4.67 ± 2.37	2.18 ± 0.65	0.944
TNFα	4.17 ± 0.89	2.84 ± 0.27	0.301
IFNα	18.21 ± 6.99	27.48 ± 13.8	0.387
MCP1	2221 ± 603.2	418 ± 217.6	0.022 *
EGF	3067 ± 1207	18748 ± 6454	0.655

**Table 6:** Biomarker characteristics. PPV—positive predictive value. NPV—negative predictive value. LR—likelihood ratio.

	AUC	Cut-Off	Sensitivity	Specificity	PPV	NPV	LR
I-TAC/CXCL11	0.77	10.49 pg/mL	66.70%	90%	92.30%	60%	6.66
CXCL10	0.71	131.50 pg/mL	53%	100%	100%	47.40%	4.71
IL6	0.73	31.82 pg/mL	63.20%	80%	85.70%	53.30%	3.15
MCP1	0.76	10,000 pg/mL	53%	100%	100%	55.60%	5.3
Blood eosinophil count	0.68	240	55%	81.82%	84.6%	50%	3.03
Urinary leukocyte count	0.47	26/µL	65%	27%	47.2%	43.8%	0.9
Model 1			84.2%	83.3%	88.9%	76.9%	5.04
Model 2			81%	91.7%	94.4%	73.3%	9.76

## Data Availability

Not applicable.

## Supplementary Materials

The following are available online at https://www.mdpi.com/article/10.3390/jcm10132986/s1, Table S1: Correlation of the degree of tubulointerstitial infiltrate with the urinary concentration of the cytokines and the analytical variables. Table S2: Value of the cytokines at diagnosis and follow-up.
