# Supplementary material for: Urinary Cytokines Reflect Renal Inflammation in Acute Tubulointerstitial Nephritis: A Multiplex Bead-Based Assay Assessment
Source: J Clin Med. 2021 Jul 4;10(13):2986. doi: 10.3390/jcm10132986 (PMC8268986; doi:10.3390/jcm10132986)
Supplement: Supplementary file 1 [file jcm-10-02986-s001.zip › jcm-1282244-supplementary.pdf]

Supplementary Table S1. Correlation of the degree of tubulointerstitial infiltrate with the urinary concentration of the cytokines and the analytical variables.

	**Spearman Rho**	***p*-Value**
**ITAC/CXCL11**	0.574	**0.001***
**MIG/CXCL9**	0.376	**0.049***
**ILB1**	0.009	0.965
**CXCL10**	0.614	**0.001***
**IL6**	0.342	0.075
**IL17**	0.398	**0.041***
**TNFalfa**	0.318	0.099
**IFN alfa**	0.403	**0.034***
**MCP1**	0.472	**0.013***
**EGF**	0.41	**0.03***
**Serum creatinine**	0.045	0.82
**Serum CRP**	0.435	**0.021***
**Urinary leukocyte count**	0.317	0.101
**Urinary erythrocyte count**	0.011	0.957
**Blood eosinophil count**	0.25	0.199

Supplementary Table S2. Value of the cytokines at diagnosis and follow-up.

	**Diagnosis sample**	**Follow-up sample**	***p*-Value**
**I-TAC/CXCL11**	44.34 ± 20.74	4.36 ± 2.05	**0.002***
**MIG/CXCL9**	1044 ± 457.3	22.86 ± 8.67	**0.007***
**IL1B**	5.8 ± 2.99	17.73 ± 15.89	0.82
**CXCL10**	441.3 ± 248.7	53.02 ± 23.83	**0.02***
**IL6**	456 ± 408.2	18.98 ± 5.72	0.1
**IL17**	11.69 ± 9.86	1.48 ± 0.36	0.15
**TNF alfa**	8.65 ± 3.81	3.55 ± 0.16	0.08
**IFN alfa**	30.63 ± 26.48	2.58 ± 1.51	0.3
**MCP1**	14106 ± 6727	6735 ± 3877	0.27
**EGF**	4461 ± 1212	11727 ± 2940	**0.016***
**Creatinine**	291.7 ± 47.67	123.8 ± 13.09	**0.002***
**CRP (mg/mL)**	113.7 ± 42.2	5.52 ± 3.21	**0.008***
**Urinary leukocyte count (cells/uL)**	756.3 ± 670.1	20 ± 12.7	0.4
**Eosinophil blood count (/10e6)**	305.5 ± 78.74	237.3 ± 58.42	0.78
